# Predicting Fluctuating Rates of Hospitalizations in Relation to Influenza Epidemics and Meteorological Factors

**DOI:** 10.1371/journal.pone.0157492

**Published:** 2016-06-16

**Authors:** Radia Spiga, Mireille Batton-Hubert, Marianne Sarazin

**Affiliations:** 1 Service de Santé publique et d’information médicale, Centre Hospitalo-Universitaire, Saint-Etienne, France; 2 Ecole Nationale Supérieure des Mines, Unité Mixte de Recherche 6158, Institut Fayol, Saint-Etienne, France; 3 Institut National de la Santé et de la Recherche Médicale, Unité Mixte de Recherche en Santé 1136, Paris, France; 4 Sorbonne Universités, Université Pierre et Marie Curie Paris 06, Paris, France; 5 Centre Ingénierie et Santé, Ecole Nationale Supérieure des Mines, Saint Etienne, France; 6 Département d’Information Médicale, Centre Hospitalier, Firminy, France; Columbia University, UNITED STATES

## Abstract

**Introduction:**

In France, rates of hospital admissions increase at the peaks of influenza epidemics. Predicting influenza-associated hospitalizations could help to anticipate increased hospital activity. The purpose of this study is to identify predictors of influenza epidemics through the analysis of meteorological data, and medical data provided by general practitioners.

**Methods:**

Historical data were collected from *Meteo France*, the Sentinelles network and hospitals’ information systems for a period of 8 years (2007–2015). First, connections between meteorological and medical data were estimated with the Pearson correlation coefficient, Principal component analysis and classification methods (Ward and k-means). Epidemic states of tested weeks were then predicted for each week during a one-year period using linear discriminant analysis. Finally, transition probabilities between epidemic states were calculated with the Markov Chain method.

**Results:**

High correlations were found between influenza-associated hospitalizations and the variables: Sentinelles and emergency department admissions, and anti-correlations were found between hospitalizations and each of meteorological factors applying a time lag of: -13, -12 and -32 days respectively for temperature, absolute humidity and solar radiation. Epidemic weeks were predicted accurately with the linear discriminant analysis method; however there were many misclassifications about intermediate and non-epidemic weeks. Transition probability to an epidemic state was 100% when meteorological variables were below: 2°C, 4 g/m^3^ and 32 W/m^2^, respectively for temperature, absolute humidity and solar radiation. This probability was 0% when meteorological variables were above: 6°C, 5.8g/m^3^ and 74W/m^2^.

**Conclusion:**

These results confirm a good correlation between influenza-associated hospitalizations, meteorological factors and general practitioner’s activity, the latter being the strongest predictor of hospital activity.

## Introduction

In France, rates of hospitalizations depend primarily on admissions from hospital emergency departments. Anticipating the flux in the number of hospitalizations is usually done daily and contextually based on empiric evidence [1]. Among the hypotheses considered to explain the observed fluctuations, two major factors appear to be the climate and epidemics. Indeed, an increase in hospitalizations is observed during epidemic peak periods of certain viral infections [2,3]. This phenomenon is observed particularly for outbreaks of influenza that occur seasonally, peaking during the winter season in temperate regions. Although benign, influenza represents a public health problem with an important morbidity and mortality rate in high risk individuals [4–7]. Despite gains in clinical knowledge and the existence of vaccines, influenza reappears every year as a seasonal epidemic with variable duration and intensity [8,9]. This phenomenon is in part explained by the characteristic of the virus: it is an enveloped RNA virus that is member of the *Orthomyxoviridae* family and genus *influenzae* of which there are three types A, B and C, and the first two are found in humans. These viruses are very unstable because of gene mutations that prevent a permanent immunization of infected individuals [10–12]. Virus A (H1N1 and H3N2) and B are primarily active during epidemics [13].

In order to explain the seasonality of the flu, two other hypotheses have been made. The first, the seasonal variation of the host’s immune status may be linked to sun exposure and the photosynthesis of vitamin D. Vitamin D plays a role in regulating acquired immunity and in reinforcing the innate immune system [14–16].

The second hypothesis is that both temperature and humidity may contribute to the transmissibility and viability of the virus. In their study on domestic guinea pigs, Lowen *et al* [17] have shown that viral transmission increases at temperatures below 5°C and in low humidity conditions. The formation and stability of air-born droplet nuclei that contain the virus are favoured in this environmental context [18]. Other studies on flu epidemic modeling and prediction have confirmed the role of temperature and absolute humidity (AH) in the spread of the virus on outpatients [19–24].

Based on these findings, the purpose of this study is to predict an influenza epidemic hospital status. This is achieved by identifying the strongest predictors, and establishing the time lags required to have an effect on hospital epidemic outbreak. To this end, two approaches may be used: the Linear Discriminant Analysis (LDA) including meteorological and outpatient data, and the Markov Chain method to calculate the transition probability from an epidemic state to another. Using these methods as predictive tools would allow a better management at a hospital level. This study was undertaken in France in the department of the Loire (Rhône-Alpes region, in East-central France), and represents a first step in the prediction of operational management of hospital emergency services.

## Materials and Methods

### Source Data

All data were collected during the period spanning the 1st week of 2007 to the 8^th^ week of 2015 and in a geographically confined territory: the southern part of the department of the Loire located in the Rhône-Alpes region of France, with 750 000 inhabitants. The average distance from the two main hospitals of the studied area is about 20 km [25]. Data were collected in aggregated form (patient number per week) and did not require approval from an ethics committee.

#### Meteorological Data

Data were taken from the Andrézieux Station managed by *Météo France* [26]. Variables considered were temperature in degrees Celsius (°C) and relative humidity in percent measured as a daily average, as well as the daily solar duration time in minutes.

#### Epidemic Data

Data for this study were obtained from the European network Sentinelles [27]. Sentinelles is a network of 1300 private practice doctors and volunteers, spread across Metropolitan France, that collects information on an on-going basis on eight health indicators, including influenza, that are encountered in daily practice. Information includes the age, sex, immunization status of patients presenting influenza-like symptoms who are seen during consultations with general practitioners. Forty-two general practitioners, dispersed evenly over the territory concerned, were involved in the collection of data. The total weekly incidence rate of influenza-like-illness (ILI) over the territory was calculated using the adjustment method for the usual general population of the Sentinelles network.

#### Hospital Data

Patient data were provided by the health record systems of the two main hospitals in the geographical zone concerned: The University-Hospital of Saint Etienne and Firminy Hospital. Previously anonymized using the official national French insurance encryption software [28,29], they were extracted in an aggregated form (weekly data). The data collected were age, sex and diagnosis coded according to the 10^th^ revision of the International Classification of Diseases (ICD-10). Three groups of patients were established:

- Influenza-associated hospitalizations (IAH): Hospitalized patients with a diagnosis of viral influenza or ILI as a primary or accompanying diagnosis, for a length of stay of less than a week. Codes and decision-making algorithms used to characterize this state were based on a previous study [30].
- Hospitalized patients admitted by emergency departments for all causes (ED admission).
- Patients who visited emergency departments, but who were not subsequently hospitalized (ED visit)

#### Variables Transformation

To coincide with the available weekly data of Sentinelles network, meteorological data were aggregated into weekly averages.

Furthermore, the absolute humidity in g/m^3^ was calculated using relative humidity according to the Clausius-Clapeyron equation [20], and solar radiation was converted to W/m^2^ using the Angström equation [31].

The hospital epidemic threshold was characterized for each week by taking the quartiles of hospitalizations associated to the influenza variable (IAH). A categorical variable was constituted taking the value 0 or non-epidemic state for case volumes below the first quartile, the value 1 or intermediate state (volumes between the 1^st^ and 2^nd^ quartile), and the value 2 or epidemic state (volumes above the 3^rd^ quartile).

### Data Analysis

Statistical analyses were used to assess the average value and variances of each of data set. The year 2009 was excluded from the analyses described below as it was a pandemic year: this study focused only on epidemic influenza. The relationship between influenza-associated hospitalizations (IAH) and other variables (meteorological, emergency departments and Sentinelles) was assessed by the Pearson correlation coefficient applying a time lag corresponding to the latent influence of meteorological and epidemic factors on hospitalizations: assuming that explicative variables effects on IAH’s are not immediate time lags have been tested to improve correlations. The Kruskal-Wallis test was used to verify the comparability between each group according to the weekly epidemic states, and according to age groups.

A principal component analysis (PCA) identified some common groups in the data and correlations between the variables. The n individuals correspond to the weeks (from the 1^st^ week of 2007 to the 8^th^ week of 2015); the p variables correspond to the meteorological, Sentinelles, and hospital data.

Two methods of classification: Ward’s hierarchical clustering then the k-means classification, where performed to obtain the maximal similarity of n individuals within clusters, and maximal dissimilarity of individual profiles between clusters [32].

The Ward’s method consists in aggregating two clusters such that the growth of within-inertia is minimum at each step of the algorithm. The within-inertia characterizes the homogeneity of a cluster. The hierarchy is represented by a dendrogram which is indexed by the gain of within-inertia. The hierarchical clustering here is performed onto the principal components. The partition obtained from the cut of the hierarchical dendrogram, is introduced as the initial partition of the K-means algorithm.

The K-means algorithm is a partitioning classification algorithm which iteratively regroups into K clusters a set of n individuals characterized by m variables. Each cluster is centred around a point, called the cluster centroid, which represents the average coordinate of the cluster’s elements. Centroids are recalculated at each iteration and these steps are repeated until the centroids no longer move.

### Predictive analysis

A Fisher’s linear discriminant analysis (LDA) made it possible to define discriminant functions and then to predict the membership group of the weeks (epidemic, intermediate or non-epidemic) based on predictor variables [33].

LDA builds j = min(*k*-1,*p*) discriminant functions that estimate discriminant scores (*D ji*) for each of *i = 1*,…,*n* individuals classified into k groups, from p linearly independent predictor variables (X) as

Dji=wi1X1i+wi2X2i+⋯+wipXpi

[*i* = 1,…,*n* and *j* = 1,…,min(*k*−1,*p*)]

Discriminant weights (*w ij*) are estimated by ordinary least squares so that the ratio of the variance within the k groups to the variance between the k groups is minimal. The classification function is:

Cji=cj0+cj1X1i+cj2X2i+⋯+cjpXpi

Each of the j = 1,…,k groups can therefore be constructed from the discriminant scores. The coefficients of the classification function for the j^th^ group are estimated from the within sum of squares matrixes (W) of the discriminant scores for each group and from the vector of the p discriminant predictors means in each of the classifying groups (M) as **C** *j* = **W** − 1**M** with ***cjo*** = **log*p*** − **12**/**C*j*M*j***

The prediction was tested on each year using as training sample the remaining six years. Then the confusion matrix determined the number of successful recognitions, and identified the incorrect match confused with another word. In general, for N number of words, the framework will generate an N × N confusion matrix (Table 1).

**Table 1:** Confusion matrix.

	TRUE VALUES			
PREDICTED VALUES	P_11_	P_12_	…	P_1N_
PREDICTED VALUES	P_21_	P_22_	…	P_2N_
PREDICTED VALUES	…	…	…	…
PREDICTED VALUES	P_N1_	P_N2_	…	P_NN_

For all *i = j*, the value of p_ij_ indicates the number of correct recognitions, while for i≠j, the value of p_ij_ indicates the confusion trend.

Finally, to predict the probability of transition from one epidemic state to another, a Markov chain model was used. This method aims to specify a system of transitions, yielding probabilistic trajectories connecting current and previous or future states [34]. Identifying the transition as a random process, the Markov dependency theory emphasizes "memoryless property" i.e. the next state of any process strictly depends on its current state but not its past sequence of states noticed over time:

P(Xn+1=xn+1/X1=x1,X2=x2,…Xn=xn)=P(Xn+1=xn+1/Xn=xn)

The Markov chain method was used for winter weeks (in December, January and February), and for different meteorological conditions. The thresholds established to classify the meteorological variables were the quartiles of their values during the winter period.

Data processing and analysis were performed using R 3.1.2 software.

## Results

### Description of the Data

**Fig 1:**
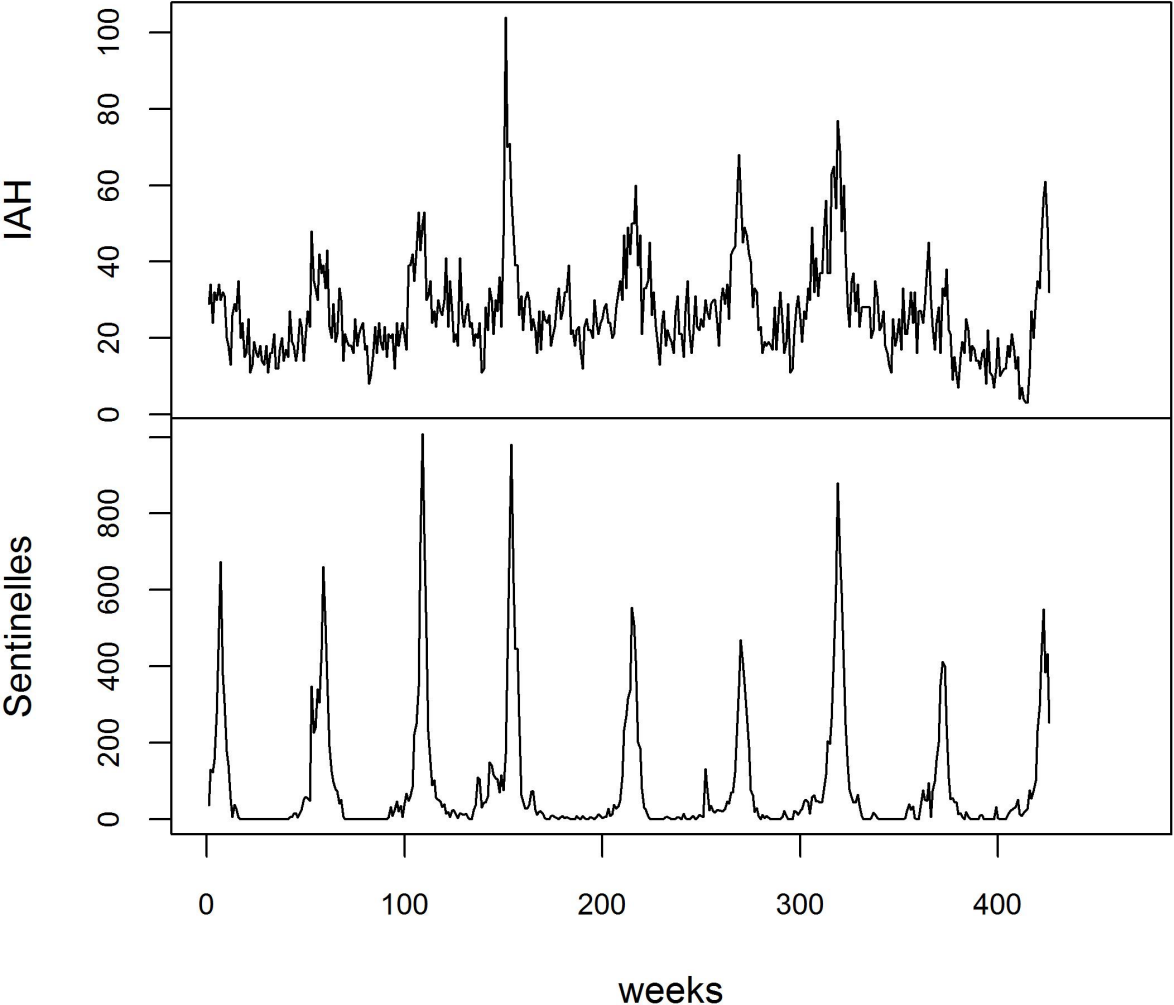
Evolution of Influenza-Associated Hospitalizations (IAH) and Sentinelles data from the first week of 2007 to 8^th^ week of 2015

**Fig 2:**
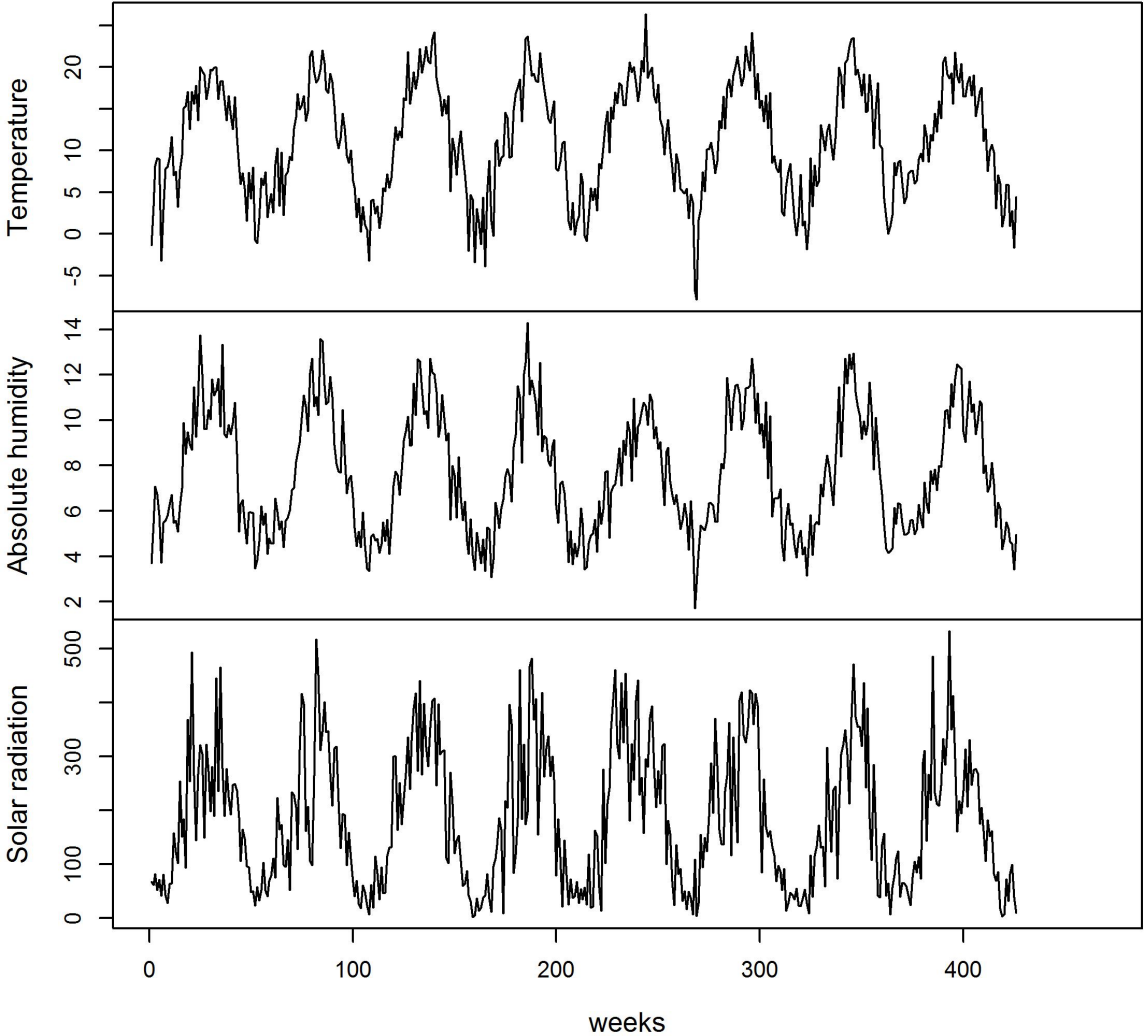
Evolution of meteorological variable values from the first week of 2007 to 8^th^ week of 2015

Between 2007 and 2015, there were 11,389 IAH with a minimum of 3 IAH /week and a maximum of 104 IAH/week (Table 2), the number of hospitalizations varied according to the years and seasons (Fig 1) with a weekly average of 31 IAH in the winter and 19 IAH for the remainder of the year, and according to age groups with a considerably larger number of people over 65 (p<0.001); During the same period the average values of temperature, absolute humidity and solar radiation were respectively 11.33°C (95% CI: 10.69–11.98), 7.68 g/m^3^ (95% CI: 7.43–7.94) and 185.80 W/m^2^ (95% CI: 173.54–198.06) in the department of the Loire (Table 2 and Fig 2).

**Table 2:** Descriptive statistics of epidemic and meteorological variables.

	Mean (%IC)	Min	max
IAH	26.73 (25.55–27.92)	3	104
ED admissions	393.5 (388.86–398.20)	258	542
ED visits	1129 (1119.48–1138.42)	887	1595
Sentinelles data	87.02 (71.61–102.54)	0	1009
Temperature (°C)	11.33 (10.69–11.98)	-7.89	26.34
Absolute humidity (g/m^3^)	7.68 (7.43–7.94)	1.71	14.29
Solar radiation(W/m^2^)	185.80 (173.54–198.06)	2.52	532.59

We found a negative correlation between IAH and meteorological factors (p<0.001), which increases when taking into account a time lag of -13 days for temperature, -12 days for absolute humidity and -32 days for solar radiation (Table 3). We also observed a close correlation between IAH and two clinical variables: hospital emergency intakes (0.53 p<0.001) and the Sentinelles data particularly when these lag by -1 week (0.70, p<0.001), (Table 3).

**Table 3:** Correlation matrix (delays in parentheses when applied).

	IAH	Sentinelles data	Sentinelles (-7days)	ED visits	ED admissions	Temperature (-13days)	Absolute humidity (-12days)	Solar radiation (-32days)
IAH	1	0.68 p<0.001	0.70 p<0.001	-0.01 p = 0.9	0.53 p<0.001	-0.63 p<0.001	-0.61 p<0.001	-0.55 p<0.001
Sentinelles data	0.68	1	0.91 p<0.001	0.02 p = 0.9	0.29 p<0.001	-0.61 p<0.001	-0.55 p<0.001	-0.50 p<0.001
Sentinelles (-7days)	0.70	0.91	1	-0.01 p = 0.9	0.27 p<0.001	-0.59 p<0.001	-0.55 p<0.001	-0.48 p<0.001
ED Visits	-0.01	0.02	-0.01	1	0.06 p = 0.9	-0.01 p = 0.9	-0.04 p = 0.9	0.00 p = 0.9
ED admissions	0.53	0.29	0.27	0.06	1	-0.39 p<0.001	-0.38 p<0.001	-0.34 p<0.001
Temperature (-13days)	-0.63	-0.61	-0.59	-0.01	-0.39	1	0.93 p<0.001	0.73 p<0.001
Absolute Humidity (-12days)	-0.61	-0.55	-0.55	-0.04	-0.38	0.93	1	0.70 p<0.001
Solar radiation (-32days)	-0.55	-0.50	-0.48	0.00	-0.34	0.73	0.70	1

**Fig 3:**
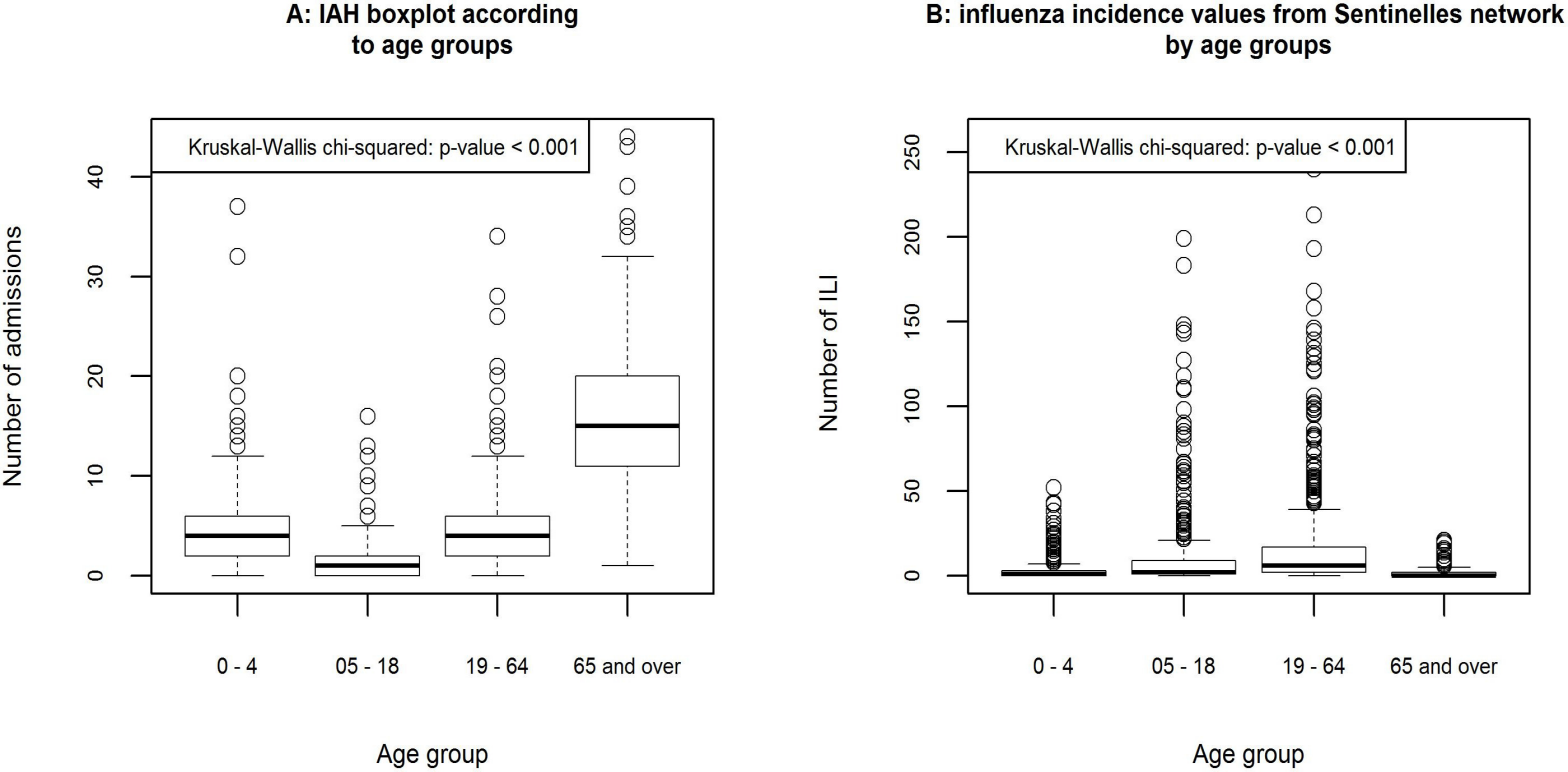
A: Influenza-Associated Hospitalizations (IAH) boxplot according to age groups; B: Boxplots of influenza incidence values (from Sentinelles network) by age groups.

The Kruskal Wallis test results showed that, among IAH cases, the 65+ age group is significantly more frequent than other age groups (p<0.001). In contrast, for influenza cases treated by general practitioners (Sentinelles data), people under 65 are significantly more preponderant (p<0.001) (Fig 3). Our analysis also showed a notable difference in the relation between the meteorological data and the presence or absence of an epidemic state in a hospital: the measured values for each meteorological variable are significantly lower during the weeks that are considered as epidemic (p<0.001).

**Fig 4:**
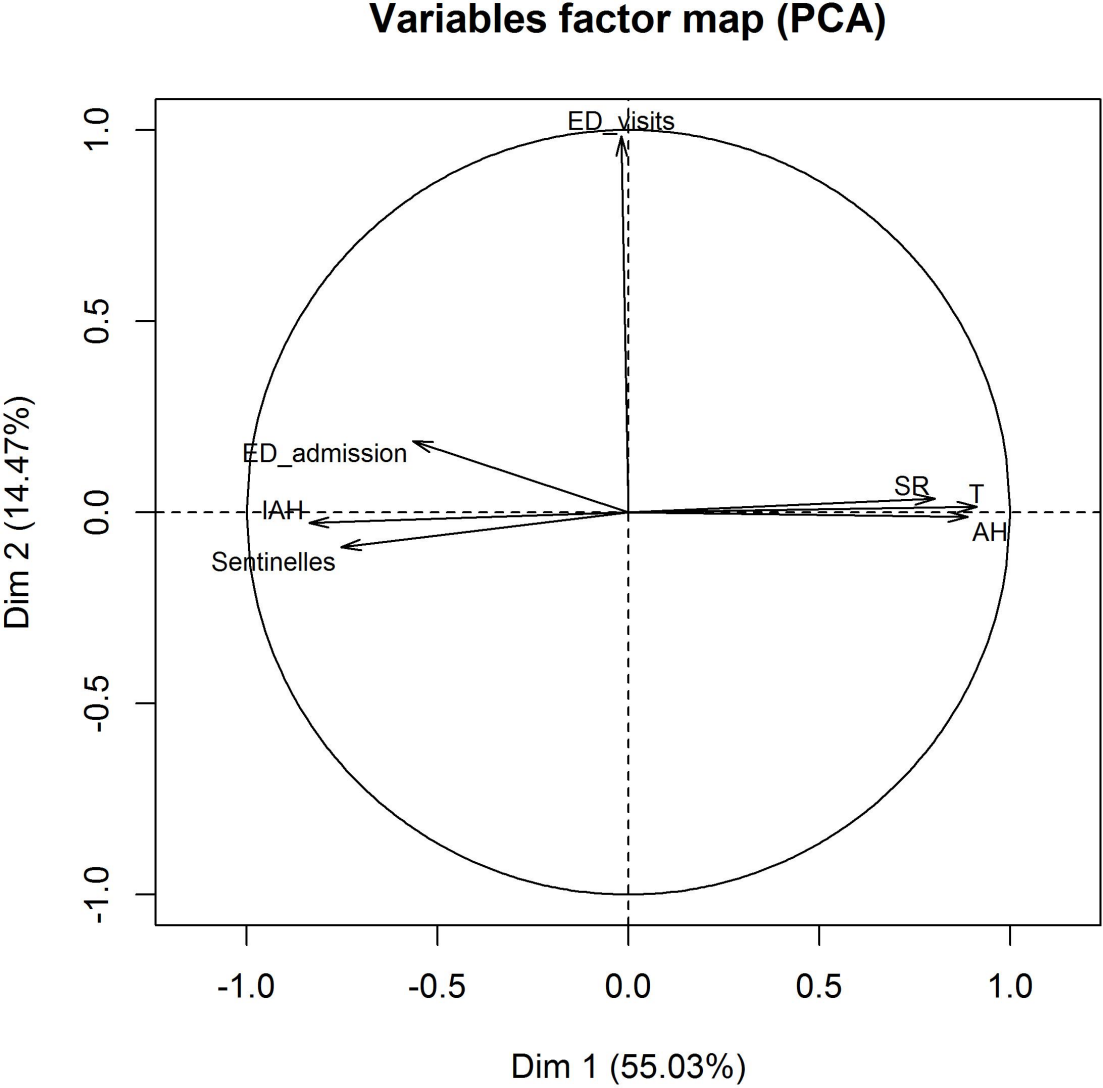
Projection of variables on the first two PCA factorials. IAH = Influenza-Associated Hospitalizations; ED = Emergency Department; AH = Absolute Humidity; SR = Solar Radiation; T = Temperature.

PCA results: the first two PCA factorial axes explain 69.5% of the information and 55.03% from axis 1 with a larger contribution from temperature and absolute humidity meteorological data (Table 4). The variables projection on the planes of the first two PCA factorial axes indicates an anti-correlation between the meteorological variables and the clinical variables (IAH, Sentinelles data and emergency data). There is no correlation between short stays and the other variables and, a non-correlation between short stays and meteorological variables (Fig 4).

**Table 4:** PCA—Contribution of variables in PCA first five dimensions.

	Dim.1	Dim.2	Dim.3	Dim.4	Dim.5
Temperature	0.91	0.01	0.24	0.18	0.2
Absolue humidity	0.89	-0.01	0.25	0.21	0.28
Solar radiation	0.8	0.04	0.22	0.25	-0.49
Sentinelles	-0.75	-0.09	-0.03	0.59	0.03
ED admissions	-0.56	0.19	0.75	-0.26	-0.01
ED visits	-0.02	0.98	-0.15	0.1	0.02
IAH	-0.84	-0.03	0.26	0.3	0.02

**Fig 5:**
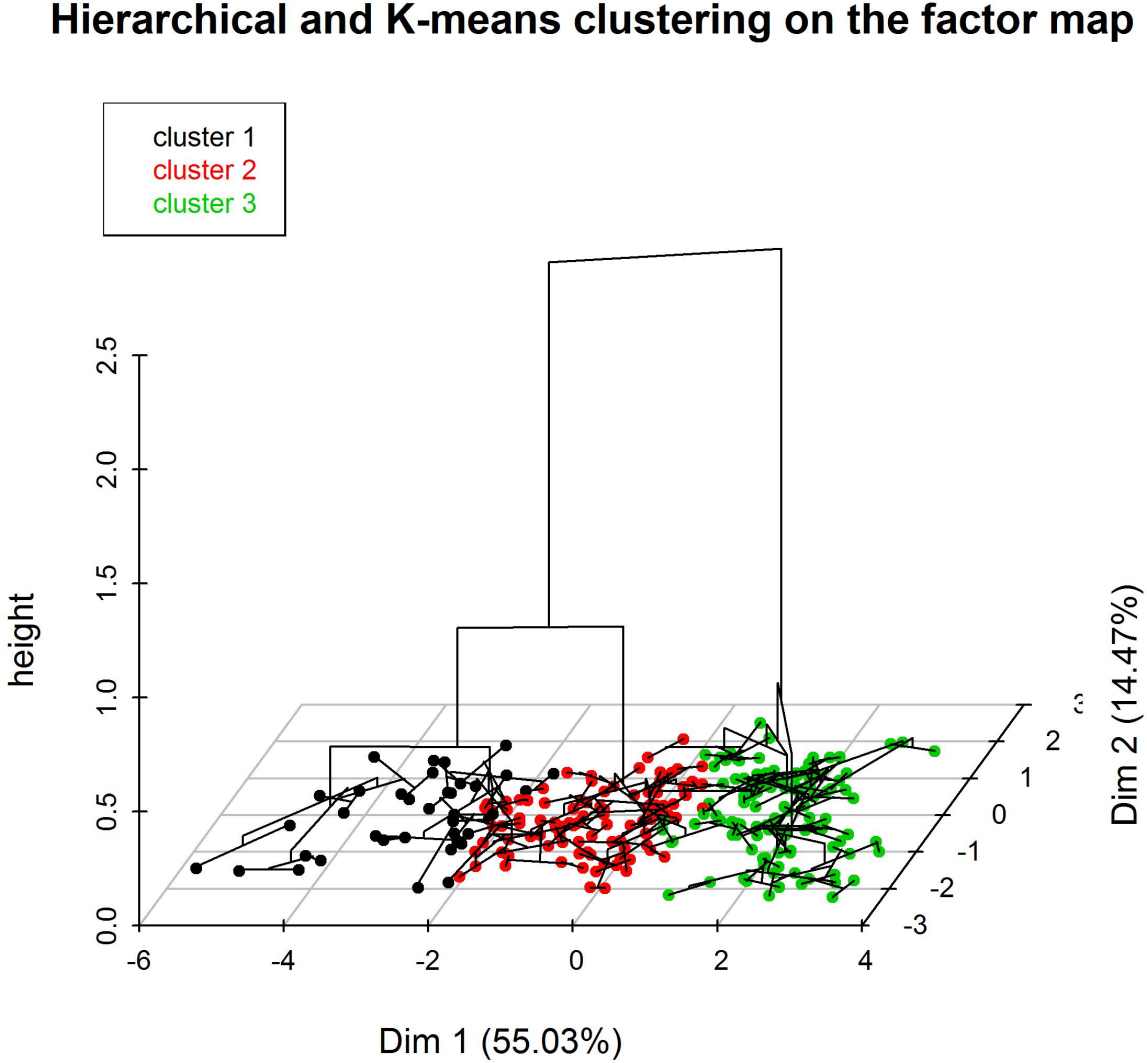
Ward’s clustering method (dendrogram) consolidated by k-means classification (colored individuals)

Using Ward’s clustering method and K-means cluster analysis the subjects could be organized into three groups (Fig 5). These groups are essentially differentiated by the temperature factor: the first group is defined by epidemic weeks with an average temperature of 2.62°C; the second group corresponds to weeks of intermediate epidemic state with an average temperature of 7.62°C; and the third group corresponding principally to non-epidemic weeks with an average temperature of 17.47°C.

### Prediction Methods

#### Linear discriminant analysis

For each year predicted, the rate of prediction error varied from 11% to 19%. The best results were obtained when the prediction was tested during 2014.

The coefficients of explanatory variables on the two LDA axes for each year, are represented in Table 5, which shows that the variable with the larger coefficient is the incidence of influenza (Sentinelles data). LDA provides correct predictions of epidemic weeks: only two epidemic weeks were wrongly classified as intermediate epidemic week in 2011 and 2012. However, according to the predicted year, misclassification numbers varied between six and ten: they corresponded in most cases to non-epidemic and intermediate epidemic weeks, in addition, for all the years considered, ten intermediate epidemic weeks, were classified as epidemic week (Table 6).

**Table 5:** LDA—Coefficients of discriminant factor son the two discriminant function axes, for each year predicted.

Predicted year	2007		2008		2010		2011		2012		2013		2014	
	[,1]	[,2]	[,1]	[,2]	[,1]	[,2]	[,1]	[,2]	[,1]	[,2]	[,1]	[,2]	[,1]	[,2]
Intercept	-0.021	-0.001	0.01	0.02	-0.03	-0.07	-0.03	-0.02	-0.01	-0.01	-0.08	0.02	-0.02	-0.00
Temperature	-0.60	-0.59	-0.38	-0.45	-0.50	-0.76	-0.64	-1.14	-0.44	0.86	-0.61	1.50	-0.43	-0.87
Solar radiation	-0.11	0.91	-0.09	1.03	-0.13	0.99	-0.07	1.03	0.01	0.39	0.08	-0.21	-0.17	0.91
Absolute humidity	0.03	0.92	-0.11	0.66	-0.08	0.96	0.04	1.17	0.08	0.01	-0.01	-0.34	-0.16	1.09
Sentinelles data	0.94	0.90	1.03	0.87	0.89	0.81	0.92	0.72	1.19	0.79	0.92	0.76	0.81	0.86

**Table 6:** Predictive LDA- Confusion matrix, for each predicted year.

		True			Error rate
Predicted		Non-epidemic	Intermediate	Epidemic	
2007	Non-epidemic	37	2	0	15.4%
2007	Intermediate	5	7	0	
2007	Epidemic	0	1	0	
2008	Non-epidemic	34	3	0	17.3%
2008	Intermediate	3	6	0	
2008	Epidemic	0	2	4	
2010	Non-epidemic	32	5	0	19.2
2010	Intermediate	5	9	0	
2010	Epidemic	0	0	1	
2011	Non-epidemic	28	4	0	17.3%
2011	Intermediate	0	8	1	
2011	Epidemic	0	4	7	
2012	Non-epidemic	19	1	0	15.4
2012	Intermediate	4	13	1	
2012	Epidemic	0	2	12	
2013	Non-epidemic	26	3	0	15.4%
2013	Intermediate	5	9	0	
2013	Epidemic	0	0	9	
2014	Non-epidemic	39	0	0	11.3%
2014	Intermediate	5	4	0	
2014	Epidemic	0	1	4	

#### Markov Chain Prediction

**Fig 6:**
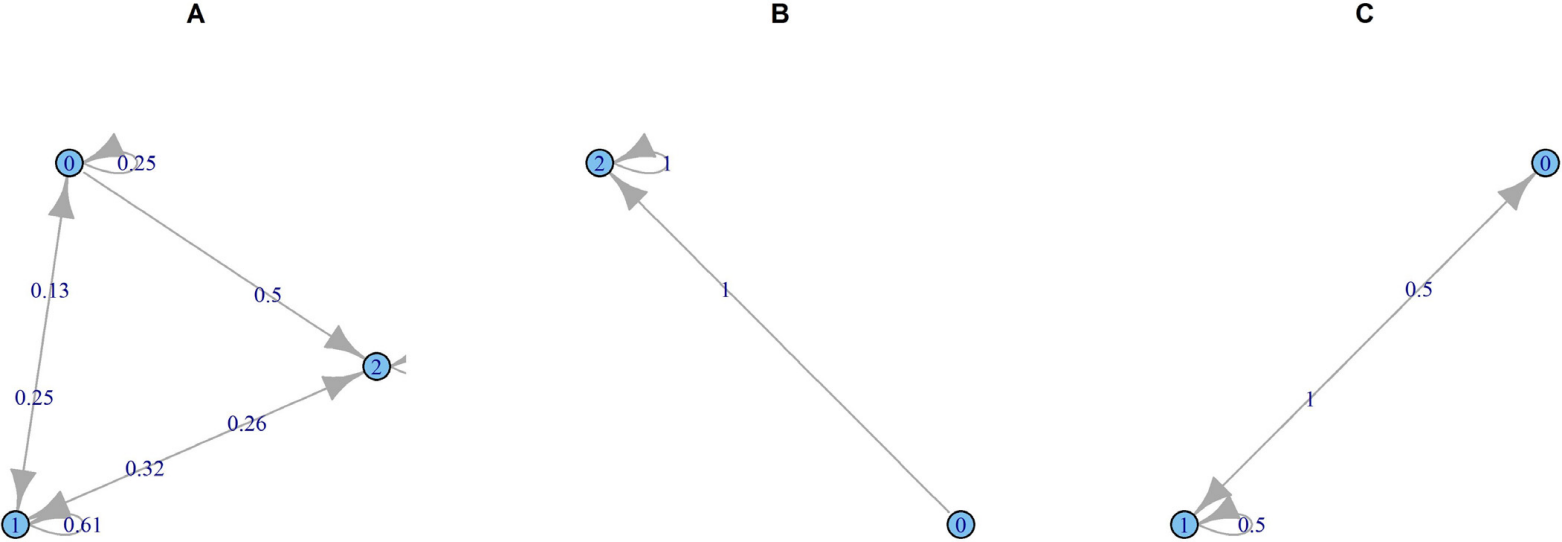
Markov Chain—A-Probability of transition for different winter epidemic states, 0 = non-epidemic, 1 = intermediate, 2 = epidemic; B—When the values of meteorological variables are below the lower thresholds: solar radiation < 32 W/m^2^, temperature < 2°C and AH < 4 g/m^3^; C- When the values of meteorological variables are above the upper threshold: solar radiation>74 W/m^2^, temperature > 6°C and AH > 5.8 g/m^3^.

Our data analysis showed that during the winter all three states can occur (epidemic, intermediate or non-epidemic) but with lower probabilities of changing to a non-epidemic week or remaining non-epidemic (Fig 6A). Probabilities of transitioning from one epidemic state to another change with the weather conditions. When the three weather variables are very low, respectively below 32 W/m^2^, 2°C and 4 g/m^3^ for solar radiation, temperature and absolute humidity, the probability of changing from a non-epidemic to an epidemic week, or remaining non-epidemic is 1 (Fig 6B). When these variables are above the highest threshold, respectively 74W/m^2^, 6°C and 5.8g/m^3^ for solar radiation, temperature and absolute humidity, the probability of changing to an epidemic week becomes nil (Fig 6C).

## Discussion

This study, carried out in France in a continental climate, reports on a new statistical method allowing to characterize the links between meteorological factors, incidence of influenza treated in non-hospital settings and hospital visits for influenza or its effects. This innovative approach based on previously published findings [17,20,35] proposes an interesting predictive method to anticipate hospital management of influenza.

The high correlation between the number of cases of influenza treated by general practitioners and the fluctuating rates of hospital emergency services entries due to influenza confirms the important role of anticipation in the management of flu epidemics. Hospitals are becoming more and more overcrowded by an influx of patients, particularly in winter and their management requires a growing number of tools to facilitate prediction of patient numbers so that the necessary human and technical resources can be adapted [36]. While the Sentinelles network’s aim is to characterize an epidemic state in the general population in France, this study proposes a novel method of prediction to allow characterization of an epidemic state within hospitals.

As described earlier, the effects of meteorological factors on flu epidemic in non-hospital and in hospital settings happen with a clinically and scientifically coherent delay. A lag of thirty-two days between sunny weather and the number of influenza-associated hospitalization can be explained by the time required for photosynthesis to occur and the release of vitamin D into the blood stream [14,37]. As to temperature and absolute humidity, these act on the virus viability and transmissibility. Even considering an incubation time of 48 hours and a latency period of a few days before consulting a physician, both related to the clinical progression of influenza, the 13 and 12 day intervals before hospitalization that were observed for these two factors appear high. However, IAH’s occur mainly among seniors (65 years and older). For this category of patients, influenza occurs later with higher risks of complication, requiring hospitalization [4,5,38]. This hypothesis is supported by the better correlation we observed when the outpatient treatment data provided by the Sentinelles network precede the IAH data by a week (Pearson correlation = 0.70, Table 3).

With the objective of predicting IAH, a first approach was to use a linear discriminant analysis at specific time intervals in order to predict the epidemic state in future weeks based on the values of explanatory variables. This method shows a good predictive accuracy to indicate epidemic weeks. The second approach examined, using Markov chains, aimed at predicting transitional states (epidemic/intermediate/non-epidemic) which are the most interesting to anticipate in terms of health planning. This method showed higher probabilities when the weather conditions are considered in the analysis; this demonstrates the usefulness of close interactions with Meteorological centres for predicting hospitalizations for hospital management purposes.

The use of these approaches is indeed possible, especially as the different data producers (Météo France and Sentinelles network) operate in such a way that each variable is provided within a time frame compatible with the application of this method. In addition, taking into consideration the week delay for Sentinelles network data supports the predictive role of such a model. Based on our results, it is possible to consider designing computerized tools for hospital use. However, this process depends on the timely availability of hospital data which remains slow in relation to the required prediction timelines and might limit the software’s effectiveness. The implementation of an individual billing plan in health establishments known as “Facturation Individuelle Des Etablissement de Santé” (FIDES) in France [39] that will soon be adapted to hospital stays, opens up the prospect of using such tools in real time.

This study has some limitations. It covers only seven epidemic events, integrating more years would reinforce the reliability of the model and provide better hindsight into the data analyses. Each year a predominant virus type may affect the clinical virulence and it would be interesting to take the virus type into consideration. In addition, other factors that could also be linked to epidemics have not been tested, including the behavioural variables such as mode of transportation, place and type of work, hand washing or immunization status of the individuals [40,41]. These elements, while difficult to collect, affect the spread of an epidemic and could reinforce the predictive accuracy of the proposed model at a population level rather than individual.

## Conclusion

The results described here highlight the delays between IAH outbreak, meteorological changes and the activities of medical practitioners. As the first phase of this study is conclusive, other studies aiming to predict the intensity and duration of epidemics by a quantitative approach can be considered. The final phase would be to propose a probabilistic model geared for hospitals which would serve as a tool to help manage anticipated fluctuations of admissions to emergency services.
